# Maintenance of zilucoplan efficacy in patients with generalised myasthenia gravis up to 24 weeks: a model-informed analysis

**DOI:** 10.1177/17562864241279125

**Published:** 2024-09-21

**Authors:** Guillemette de la Borderie, Damien Chimits, Babak Boroojerdi, Melissa Brock, Petra W. Duda, Fiona Grimson, Paul Mahoney, Foteini Strimenopoulou, Gary Cutter, Inmaculada Aban, Susanna Brauner, Malin Petersson, James F. Howard, Nathan Bennett

**Affiliations:** UCB, 420 rue d’Estienne d’Orves, Colombes 92700, France; UCB, Colombes, France; UCB, Monheim, Germany; UCB, Morrisville, NC, USA; UCB, Cambridge, MA, USA; UCB, Slough, UK; UCB, Slough, UK; UCB, Slough, UK; Department of Biostatistics, The University of Alabama at Birmingham School of Public Health, Birmingham, AL, USA; Department of Biostatistics, The University of Alabama at Birmingham School of Public Health, Birmingham, AL, USA; Department of Clinical Neuroscience, Karolinska Institutet, Stockholm, Sweden; Department of Neurology, Karolinska University Hospital, Stockholm, Sweden; Department of Clinical Neuroscience, Karolinska Institutet, Stockholm, Sweden; Department of Neurology, The University of North Carolina at Chapel Hill, Chapel Hill, NC, USA; UCB, Morrisville, NC, USA

**Keywords:** Bayesian, C5, complement inhibitor, external comparator, meta-regression analysis, model-informed analysis, myasthenia gravis, zilucoplan

## Abstract

**Background:**

Clinical efficacy of zilucoplan has been demonstrated in a 12-week, placebo-controlled, phase III study in patients with acetylcholine receptor autoantibody-positive generalised myasthenia gravis (gMG). However, placebo-controlled zilucoplan data past 12 weeks are not available.

**Objectives:**

Predict the treatment effect of zilucoplan versus control (placebo or standard of care) in patients with gMG up to 24 weeks.

**Design:**

A model-informed analysis (MIA) within a Bayesian framework.

**Methods:**

Part 1 of the MIA comprised a control meta-regression using aggregate data on control response over time from randomised studies and a national myasthenia gravis (MG) registry. In Part 2, a combined Bayesian analysis of individual patient-level data from the phase II (NCT03315130), RAISE (NCT04115293) and RAISE-XT (NCT04225871) studies of zilucoplan was conducted using posterior distributions from Part 1 as informative priors. Population mean treatment effect in the change from baseline (CFB) at week 24 in MG-Activities of Daily Living (MG-ADL) and quantitative MG (QMG) scores for zilucoplan versus control were assessed.

**Results:**

At week 24, the predicted mean CFB in MG-ADL score was −4.55 (95% credible interval: −6.04, −3.13) with zilucoplan versus −2.00 (−3.35, −0.64) with control (difference: −2.55 [−3.76, −1.40]). The probability of a favourable treatment effect as measured by MG-ADL score at week 24 with zilucoplan versus control was >99.9%. There was an 82.8% probability that the difference in the predicted mean CFB in MG-ADL score at week 24 was greater than the clinically meaningful threshold (⩾2.0-point improvement). Comparable results were observed with QMG.

**Conclusion:**

This MIA demonstrates the maintenance of efficacy with zilucoplan versus control up to 24 weeks. Through combining real-world evidence with data from randomised studies, this novel method to estimate long-term treatment efficacy facilitated reduced exposure to placebo in the phase III RAISE study. This methodology could be used to reduce the length of future placebo-controlled studies.

## Introduction

Myasthenia gravis (MG) is a rare, chronic, autoimmune disease characterised by fluctuating muscle weakness and fatigability, which worsen with activity.^1,2^ Many patients have disease exacerbations with conventional therapies.^3^ Exacerbations can be life-threatening, with a small subset of patients experiencing respiratory muscle weakness progressing to myasthenic crisis.^1^ Furthermore, approximately 10%–30% of patients with MG have an inadequate response to conventional treatments or experience intolerable side effects.^1,3–5^

In MG, autoantibodies bind to acetylcholine receptors (AChRs) or functionally related molecules at the postsynaptic membrane of the neuromuscular junction (NMJ) resulting in impaired synaptic transmission.^1,6,7^ One of the mechanisms by which AChR autoantibodies disrupt neurotransmission at the NMJ is through the activation of complement^1,2^ and subsequent assembly of the membrane attack complex. The terminal complement complex causes architectural damage to the postsynaptic membrane of the NMJ, resulting in impaired synaptic transmission.^2^

Zilucoplan is a small (3.5 kDa), 15-amino acid macrocyclic peptide that targets complement component 5 of the complement cascade.^6,8^ The clinical development programme investigating the use of zilucoplan in patients with AChR autoantibody-positive generalised MG (gMG) includes a double-blind phase II study, a double-blind phase III study and an open-label extension (OLE) study.^9–11^ In the successful 12-week phase II study (ClinicalTrials.gov identifier: NCT03315130), a clinical effect was observed with zilucoplan at week 4 and sustained through to week 12.^10^ Therefore, a 12-week study duration was considered appropriate and sufficient to demonstrate clinical efficacy in a phase III study,^9^ while also minimising exposure to placebo. In the phase III RAISE study (ClinicalTrials.gov identifier: NCT04115293), zilucoplan demonstrated clinically meaningful and statistically significant improvements in MG-specific outcomes compared with placebo at week 12.^7,9^ Least squares (LS) mean difference versus placebo at week 12 Myasthenia Gravis Activities of Daily Living (MG-ADL) score (95% confidence interval [CI]) was −2.09 (−3.24 to −0.95); *p* = 0.0004.^9^

Patients who completed either the phase II or phase III study and wished to continue receiving zilucoplan could enrol in the ongoing OLE study, RAISE-XT (ClinicalTrials.gov identifier: NCT04225871).^11^ Data from an interim analysis of RAISE-XT have shown that efficacy is sustained with zilucoplan through 60 weeks.^11^ Due to the open-label nature of this study, placebo-controlled zilucoplan data past 12 weeks were not available.

While randomised controlled studies represent the gold standard for demonstrating the clinical efficacy of investigational treatments, concerns have been raised about prolonged exposure to a placebo.^12^ Nevertheless, other complement inhibitors approved for the treatment of gMG have pivotal study durations of up to 26 weeks, making it of interest to predict the treatment effect of zilucoplan versus placebo (control) for a similar treatment duration.^13,14^ Therefore, a model-informed analysis (MIA) within a Bayesian framework for up to 24 weeks was pre-specified prior to unblinding the phase III RAISE study.

A Bayesian statistical approach can be used to make inferences about treatment effects by providing a formal mathematical method for the combination of existing, or historical, external data with data from a current clinical study. One possible outcome of this approach is augmentation of the current clinical study data.^15,16^ Patient registries, real-world evidence and completed randomised controlled studies represent commonly used sources of historical data to construct a ‘prior’ distribution for inclusion in Bayesian analyses.^15–17^ Via the use of this prior, Bayesian analysis allows for borrowing strength from the data sources external to the current observed clinical study data.^16,17^ This method has the potential to reduce the number of control patients required in a placebo-controlled study and the length of patients’ exposure to placebo, thereby reducing the time taken to bring novel therapeutic options to patients while avoiding unnecessary placebo exposure.^15,16^ The prior can be informative (historical) or non-informative (vague).^16,18^ Unlike the informative prior, which is based on historical data, the non-informative prior is not supplemented by historical external data,^15,16,18^ and therefore, the Bayesian inference would not be influenced by prior beliefs regarding the placebo effect.

The pre-specified MIA leveraged relevant information from three external data sources, which included real-world evidence from the national Swedish MG Registry, data from The Thymectomy Trial in Non-Thymomatous Myasthenia Gravis Patients Receiving Prednisone Therapy (MGTX; ClinicalTrials.gov identifier: NCT00294658) and data from a systematic literature review (SLR) of completed randomised controlled studies.

The national Swedish MG Registry was launched in 2011 based on a local registry in Stockholm. The registry is voluntary, comprising 1709 patients, of whom 1422 are actively followed routinely. The primary focus of the registry is the inclusion of patients who have been diagnosed within the last 10 years, as well as patients receiving immunosuppressant therapy, with an estimated coverage of >44% of the whole MG population based on a recent prevalence estimate and the total Swedish population.^19,20^ The registry has been used in cohort studies to determine real-world patient outcomes in Sweden.^21^ MGTX was a multicentre, rater-blinded, randomised study conducted between June 2006 and December 2015 to compare extended transsternal thymectomy combined with prednisone against prednisone alone in patients with non-thymomatous MG. Patients were followed for a minimum of 3 years.^22^ Data from these two external sources were used together with the SLR of completed randomised controlled studies in a control meta-regression (including placebo and standard of care data) to build an informative prior.

Here, we report the results of a Bayesian individual combined analysis of zilucoplan clinical study data using this informative prior to estimate the maintenance of efficacy of zilucoplan versus an external ‘control’ group for up to 24 weeks. This analysis was designed to extend the results observed in the 12-week phase III RAISE study in lieu of placebo-controlled data past 12 weeks. The use of non-informative priors was evaluated through sensitivity analyses.

## Methods

Full methodology of this MIA is available in Supplemental Appendix 1. Briefly, external control data were chosen based on the selection of a patient population comparable to the RAISE clinical study population. All analyses are reported for the zilucoplan 0.3 mg/kg group, the dose used in the phase III RAISE study and the control (placebo or standard of care) group, which is similar to the placebo group in the phase III RAISE study.

### Data sources

#### Summary data from an SLR

An SLR was conducted between 24 September 2019 and 27 January 2022. The search strategy was designed to identify randomised, placebo-controlled studies similar to the phase III RAISE study. Studies were identified using Embase^®^, MEDLINE^®^, MEDLINE^®^ In-Process and the Cochrane Central Register of Controlled Trials (CENTRAL). Search terms were based on words characterising the disease and the study design (Supplemental Table 1). Two independent reviewers screened the titles and abstracts of the records identified in the database search based on pre-defined inclusion criteria; full-text articles of relevant abstracts were then retrieved and reviewed. Any discrepancies in their decisions were resolved by a third, independent reviewer. Conferences not indexed in the Embase® database were hand-searched via the respective conference websites to identify relevant conference proceedings and abstracts not published in journals as full-text articles from 2017 to 2022. Bibliographic searching of systematic reviews and meta-analyses was conducted to identify any missing studies; ClinicalTrials.gov and the EU Clinical Trials Register were also searched. After completion of the SLR, ravulizumab data were added following their publication in April 2022.^13^ The MIA included randomised, placebo-controlled studies in patients aged ⩾18 years with moderate-to-severe gMG, in which baseline MG-ADL score and at least one post-baseline MG-ADL score up to week 28 were available (Supplemental Table 2). The same approach was taken for quantitative MG (QMG) scores.

Values of the LS means of the change from baseline (CFB) derived from a statistical model or, if not available, the mean CFB, and associated standard errors from the placebo treatment groups were sourced from identified studies. These data at all available visits up to week 28 were extracted to capture the nearest timepoint to week 24, accounting for the visit schedule of each identified study.

#### Individual patient-level data

Following a literature review and an assessment of known clinical databases and studies in MG, two suitable sources in which individual patient-level data could be shared were identified for inclusion in the MIA: the Swedish MG Registry and the MGTX study.^22^

The selection of patient cohorts was based on the main inclusion and exclusion criteria of the phase III RAISE study. Inverse probability treatment weighting (IPTW) using odds weighting methodology adjusted on MG-ADL and QMG score at baseline was applied to achieve enhanced comparability to RAISE study patients. The inclusion criteria by which patient cohorts were selected for both external individual patient-level data sources are presented in Supplemental Table 2.

The MIA applied censoring to patients from the Swedish MG Registry for 6 months post-thymectomy and 4 weeks following rescue therapy, defined as the receipt of intravenous or subcutaneous immunoglobulin, or plasma exchange. Censoring was also applied by the model to the prednisone and thymectomy groups from the MGTX study to identify two separate groups for inclusion (Supplemental Table 2): the prednisone group, which included all patients randomised to prednisone monotherapy in the MGTX study with censoring of the first 3 months to remove the period where the largest number of steroid dose changes occurred, and the thymectomy group, which included all patients randomised to thymectomy in the MGTX study with censoring of the first 12 months to reduce the effect of thymectomy. The censoring was intended to reflect the inclusion and exclusion criteria of the phase III RAISE study,^9^ while considering the specificity of each data source. Due to the real-world nature of the Swedish MG Registry data and based on clinical practice, a censoring period of 6 months post-thymectomy was deemed appropriate.

Prior to RAISE, the LS mean changes from baseline up to week 12 in MG-ADL scores and the associated 95% CIs for each of the two groups identified from the MGTX study were compared visually to LS means and 95% CIs of the placebo treatment group from the phase II zilucoplan study. Both groups from the MGTX study showed similar LS mean changes from baseline in MG-ADL scores to those for the placebo group in the phase II study (data not shown), and therefore, the two MGTX study groups were naïvely pooled into a single group for inclusion in the MIA.

#### Zilucoplan clinical studies

A description of the zilucoplan clinical studies used within this MIA is presented in Supplemental Table 3.^9–11^

### Study outcomes

The population mean treatment effect with respect to the CFB at week 24 in MG-ADL and QMG scores were assessed for the zilucoplan and control groups. Predicted mean changes from baseline and associated two-sided 95% credible intervals (CrIs), which are Bayesian analogues to CIs in frequentist analyses,^23^ through week 24 in MG-ADL and QMG scores were also calculated for the zilucoplan and control groups. The distribution of the difference in predicted mean CFB to week 24 between the two groups (zilucoplan minus control) was generated.

The posterior probability that the predicted mean CFB in MG-ADL score at week 24 would be lower with zilucoplan versus the control was also calculated. Additionally, the posterior probability that the difference in predicted mean CFB in MG-ADL score between zilucoplan and the control at week 24 was lower than the threshold of a ⩾2.0-point reduction was generated. The same outcomes were assessed for QMG scores, but using a threshold of a ⩾3.0-point reduction. These thresholds were applied based on the clinically meaningful thresholds for MG-ADL and QMG scores defined in the literature.^24,25^ The posterior probability can be interpreted directly as the probability that there is an improved treatment effect with zilucoplan versus the control.^16^ The posterior probability that there is a worsened or similar treatment effect with zilucoplan versus the control, defined as *p*, was also calculated. This is not conceptually equivalent to the frequentist *p*-value.^16^

### Statistical methods

This MIA, including the selection of data used for the control group, was pre-planned and finalised prior to RAISE study unblinding. Full statistical methods are available in Supplemental Appendix 1.

Sample size calculations for the phase II study and RAISE study have been reported previously.^9,10^ Briefly, for the RAISE study it was calculated that a target sample size of 156 patients (88 patients per group) would provide approximately 94% power to detect a LS mean difference in MG-ADL score of 2.3 (standard deviation 3.7) between the zilucoplan and placebo treatment groups, using a two-sided alpha of 5% and assuming an attrition rate of 15%. As this MIA is a meta-analysis of the phase II and RAISE data, plus aggregate external summary data (SLR, Swedish MG Registry and MGTX study), the sample size includes 101 patients in the zilucoplan group and 488 patients in the control group. The latter comprises 103 patients from the placebo groups of the phase II study and the RAISE study, and 385 patients from the aggregate external data after odds weighting.

This analysis within a Bayesian framework consisted of two parts. Part 1 synthesised information based on the aggregate external summary data on response to the control over time through a meta-regression model. Aggregate external summary data for the Swedish MG Registry and MGTX study were obtained using a mixed model with repeated measurements (MMRM) with time as a fixed effect (categorical) and baseline as a covariate using an unstructured correlation structure and weighted according to IPTW. Weights were used to achieve better similarity between the populations from the Swedish MG Registry and MGTX study and the phase III RAISE study in terms of disease severity (MG-ADL and QMG scores at baseline). These data were combined with data extracted from the SLR. Part 2 was a combined Bayesian analysis of individual patient-level data from the double-blind studies (phase II and RAISE) and OLE study (RAISE-XT), which used the posterior distributions resulting from the analysis in Part 1 as informative priors, down-weighted by 30% to avoid prior-data conflict. The choice of the percentage by which to down-weight the priors in the main analysis was arbitrary, and a sensitivity (tipping point) analysis was performed to evaluate the impact of different weightings on the results. Analyses assumed a linear model with log time in Part 1 and Part 2. This model included log time because it assumed a response in the first weeks followed by a sustained effect as seen in the literature for MG-ADL score.^9,13,14^ The same model was chosen to evaluate QMG scores, as their evolution over time is comparable to that of MG-ADL scores.^26^ An Emax model was utilised in a supplementary analysis to capture a plateau of response at earlier time points.

The primary analysis utilised the modified intention-to-treat (mITT) population in Part 2, which included all randomised patients in the double-blind studies (phase II and RAISE) who received at least one dose of zilucoplan or matched placebo and had at least one post-dosing MG-ADL score during the 12-week double-blind period. In addition, data from the ongoing OLE study (RAISE-XT) up to week 24 were used for patients randomised to zilucoplan in the double-blind studies using a data cut-off date of 8 September 2022. The same imputation method that was pre-specified in RAISE^9^ was also used for the zilucoplan clinical studies (phase II, RAISE and RAISE-XT) for this MIA. Briefly, use of rescue therapy, an adverse event of myasthenic crisis or death were defined as intercurrent events and assumed as a treatment failure. Any data after the first intercurrent event were imputed as baseline or the last available score, whichever was worse. Any other monotone missing data were assumed to be missing at random.^9^

In addition to the tipping point analysis and supplementary analysis, the primary analysis was supported by a range of further sensitivity analyses that differed in the inclusion or exclusion of OLE data for zilucoplan, the use of an informative (historical) or non-informative (vague) prior and the analysis set used. Two sensitivity analyses are reported here. Sensitivity analysis 1 was performed using the mITT_RAISE_ population in Part 2, which included all randomised patients in RAISE (only) who received at least one dose of zilucoplan or matched placebo and had at least one post-dosing MG-ADL score during the 12-week double-blind period. Data from RAISE-XT were not included in Part 2. Data from the placebo group of the phase II study were included in Part 1. Sensitivity analysis 2 utilised non-informative (vague) priors in Part 2.

## Results

### Data sources and patients

Based on the randomised controlled studies identified in the SLR, 42 studies were considered based on their study populations (Supplemental Figure 1). A total of six studies with MG-ADL data and nine studies with QMG data at baseline and for at least one time point up to week 28 were included. Swedish MG Registry data were available from 1200 patients across 8 clinics. For the analysis of MG-ADL and QMG scores, 16 and 15 of these patients respectively met the selection criteria. The MGTX study enrolled 126 participants from 70 sites, of whom 53 met the selection criteria for the analysis of MG-ADL score and 62 patients met the selection criteria for the analysis of QMG score. Baseline characteristics of the patients included in the MIA are presented in Table 1.

**Table 1.** Baseline demographics and disease characteristics of patients included in the MIA.

Category	Placebo (phase II and RAISE), *N* = 103	Zilucoplan (phase II and RAISE), *N* = 101	External data before odds weighting,^a^ *N* = 387	External data after odds weighting,^a^ *N* = 385
Age, years, mean (SD)	52.6 (15.7)	52.9 (14.6)	48.5 (15.4)	48.1 (16.1)
Sex, male, *n* (%)	45 (43.7)	39 (38.6)	158 (40.8)	154 (40.0)
Duration of disease, years, mean (SD)	8.8 (10.0)	9.2 (9.3)	7.3 (7.4)	7.4 (7.5)
MG-ADL score, mean (SD)	10.6 (3.5)	9.9 (2.5)	8.2 (2.4)	8.5 (2.8)
QMG score, mean (SD)	19.3 (4.4)	18.8 (3.8)	14.8 (4.4)	15.4 (5.6)
Geographic region, *N*_obs_^b^	103	101	16	16
North America, *n* (%)	61 (59.2)	60 (59.4)	0	0
Europe, *n* (%)	33 (32.0)	34 (33.7)	16 (100.0)	16 (100.0)
East Asia, *n* (%)	9 (8.7)	7 (6.9)	0	0
Missing, *n* (%)	0	–	–	–
Prior thymectomy, *N*_obs_^b^	103	101	239	237
Yes, *n* (%)	42 (40.8)	52 (51.5)	96 (40.2)	96 (40.5)
Prior MG crisis, *N*_obs_^b^	103	101	100	100
Yes, *n* (%)	32 (31.1)	30 (29.7)	14 (14.0)	14 (14.0)
Race, *N*_obs_^b^	103	101	235	235
White, *n* (%)	74 (71.8)	77 (76.2)	175 (74.5)	175 (74.5)
Asian, *n* (%)	15 (14.6)	8 (7.9)	39 (16.6)	39 (16.6)
Black, *n* (%)	9 (8.7)^c^	9 (8.9)	10 (4.3)	10 (4.3)
Missing, *n* (%)	3 (2.9)	7 (6.9)	5 (2.1)	5 (2.1)
Other, *n* (%)	2 (1.9)	0	6 (2.6)	6 (2.6)

Data for patients included in the MIA for the analysis of MG-ADL scores only. Baseline demographics and disease characteristics of patients included in the MIA for the analysis of QMG scores were similar, but not reported here.

a External data includes placebo-treated patients from studies of belimumab (NCT01480596), eculizumab (NCT01997229), tacrolimus (NCT01325571), ravulizumab (NCT03920293), efgartigimod (NCT03669588) and rozanolixizumab (NCT03052751)^13,14,27–30^ and data from the Swedish MG Registry and MGTX study (NCT00294658).^22^ IPTW using odds weighting adjusted on MG-ADL and QMG score at baseline was used. Due to missing MG-ADL or QMG scores, two patients from the MGTX study were excluded from the external data after odds weighting group.

b Number observed only includes studies in which data were reported.

c Black or African American.

IPTW, inverse probability treatment weighting; MG, myasthenia gravis; MG-ADL, myasthenia gravis activities of daily living; MGTX, The Thymectomy Trial in Non-Thymomatous Myasthenia Gravis Patients Receiving Prednisone Therapy; MIA, model-informed analysis; QMG, quantitative myasthenia gravis; SD, standard deviation.

Upon pooling of the external control data, no major changes in MG-ADL scores were seen between weeks 12 and 24, consistent with a maintenance phase, thereby supporting the underlying assumptions of the linear model with log time used in the MIA (Supplemental Figure 2).

### Evolution of predicted mean CFB up to week 24 in MG-ADL and QMG scores

**Figure 1.**
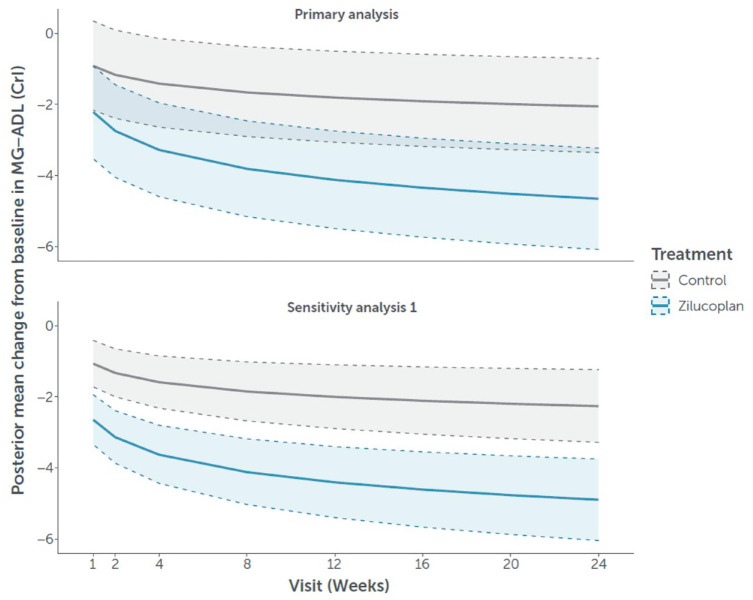
Evolution of predicted mean change from baseline (and associated 95% CrI) in MG-ADL score through week 24. Primary analysis: Data from the double-blind studies, including RAISE-XT data for patients randomised to zilucoplan in the double-blind studies, were included in Part 2. A linear model with log time using the historical prior. Sensitivity analysis 1: Placebo data from the phase II study were included in Part 1. Data from RAISE only were included in Part 2. A linear model with log time using the historical prior. CrI, credible interval; MG-ADL, myasthenia gravis activities of daily living.

The evolution of predicted mean CFB over time up to week 24 in MG-ADL score for the zilucoplan and control groups is displayed in Figure 1.

Predicted mean CFB in MG-ADL score improved rapidly in both the control and zilucoplan groups in the first 4 weeks. Throughout the 24 weeks, minimal overlap was observed between the 95% CrIs of the predicted mean CFB in MG-ADL score for the zilucoplan and control groups. In the control group, CFB slowly improved up to week 12, as observed in the double-blind studies and the external data sources. A similarly slow improvement over time was observed from week 12 to week 24 (Table 2). In the zilucoplan group, predicted mean CFB continued to improve from week 4 through week 12, and further to week 24 (Table 2). The distribution of the predicted mean CFB at week 24 for the zilucoplan and control groups is presented in Supplemental Figure 3. A graph depicting the difference between the predicted mean CFB in MG-ADL scores for the zilucoplan and control groups versus the frequentist inference is presented in Supplemental Figure 4.

**Table 2.** Predicted and LS mean change from baseline in MG-ADL and QMG scores for the MIA, RAISE and RAISE-XT studies.

Predicted mean change from baseline (95% CrI) in the MIA^a^	Zilucoplan	Control	Difference zilucoplan versus control
Primary analysis (mITT)			
MG-ADL score			
Week 12	−4.05 (−5.47, −2.69)	−1.78 (−3.08, −0.48)	−2.27 (−3.30, −1.29)
Week 24	−4.55 (−6.04, −3.13)	−2.00 (−3.35, −0.64)	−2.55 (−3.76, −1.40), *p* < 0.0001^b^
QMG score			
Week 12	−5.63 (−6.94, −4.35)	−2.56 (−3.64, −1.45)	−3.07 (−4.23, −1.90)
Week 24	−6.24 (−7.66, −4.84)	−2.92 (−4.08, −1.72)	−3.32 (−4.69, −1.95), *p* < 0.0001^b^
Sensitivity analysis 1 (mITT_RAISE_)			
MG-ADL score			
Week 12	−4.41 (−5.40, −3.40)	−2.00 (−2.90, −1.10)	−2.40 (−3.70, −1.14)
Week 24	−4.90 (−6.05, −3.75)	−2.27 (−3.28, −1.24)	−2.63 (−4.14, −1.15), *p* = 0.0002^b^
QMG score			
Week 12	−6.24 (−7.48, −4.98)	−3.04 (−4.03, −1.99)	−3.20 (−4.73, −1.70)
Week 24	−6.85 (−8.28, −5.41)	−3.43 (−4.56, −2.25)	−3.42 (−5.19, −1.68), *p* < 0.0001^b^
LS mean change from baseline (95% CI) in the RAISE study (mITT_RAISE_)^c,9^	Zilucoplan (*n* = 86)	Placebo (*n* = 88)	Difference zilucoplan versus placebo
MG-ADL score			
Week 12	−4.39 (−5.28, −3.50)	−2.30 (−3.17, −1.43)	−2.09 (−3.24, −0.95), Frequentist *p*-value = 0.0004
QMG score			
Week 12	−6.19 (−7.29, −5.08)	−3.25 (−4.32, −2.17)	−2.94 (−4.39, −1.49), Frequentist *p*-value < 0.0001
LS mean change from baseline (95% CI) in a post hoc analysis of the RAISE-XT study (mITT_RAISE_)^d^	Zilucoplan (*n* = 86)	–	
MG-ADL score			
Week 12	−4.31 (−5.42, −3.21)	–	
Week 24	−4.92 (−6.24, −3.61)	–	

a Bayesian MIA using a historical prior. In sensitivity analysis 1, phase II placebo data were used in the control meta-regression to build the historical prior.

b *p* is the posterior probability that there is a worsened or similar treatment effect with zilucoplan versus the control.

c Frequentist inference. Treatment group differences in change from baseline at week 12 for the MG-ADL and QMG scores were estimated using a linear mixed model of repeated measures analysis of covariance.^9^

d Scores after rescue or any discontinuation were imputed as treatment failure. Death was imputed as the worst possible score (post hoc conservative analysis).

CI, confidence interval; CrI, credible interval; LS, least squares; MG-ADL, myasthenia gravis activities of daily living; MIA, model-informed analysis; mITT, modified intention-to-treat; QMG, quantitative myasthenia gravis.

**Figure 2.**
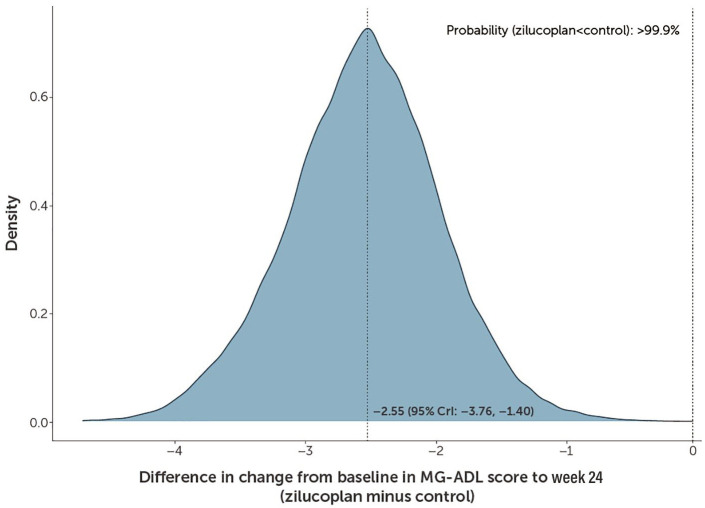
Distribution of the difference between zilucoplan and control in predicted mean changes from baseline to week 24 in MG-ADL score. A negative value in the difference (zilucoplan minus control) corresponds to an improvement in MG-ADL score. Primary analysis: Data from the double-blind studies, including RAISE-XT data for patients randomised to zilucoplan in the double-blind studies, were included in Part 2. A linear model with log time using the historical prior. CrI, credible interval; MG-ADL, myasthenia gravis activities of daily living.

The mean estimation of a population treatment effect, that is, the difference in MG-ADL score between the zilucoplan and control groups was −2.27 (95% CrI: −3.30, −1.29; Table 2) at week 12. At week 24, the mean estimation of a population treatment effect was −2.55 (95% CrI: −3.76, −1.40), translating to an improvement in MG-ADL score of 2.55 for zilucoplan versus the control (Table 2). The distribution of the difference between the zilucoplan and control groups in the predicted mean CFB to week 24 in MG-ADL score is displayed in Figure 2.

The posterior probability of an improved treatment effect with zilucoplan versus control as measured by mean CFB in MG-ADL score at week 24 was >99.9% (*p* < 0.0001; Figure 2). Further, there was an 82.8% probability that the difference in the predicted mean CFB in MG-ADL score at week 24 between zilucoplan and the control group was greater than the clinically meaningful threshold (⩾2.0-point reduction). A sensitivity analysis of the evolution of predicted mean CFB over time up to week 24 in MG-ADL score for the zilucoplan and control groups confirmed the results of the primary analysis (Figure 1 and Table 2). The results of a supplementary analysis, in which an Emax model was employed for the primary analysis instead of assuming a log-linear relationship between the CFB in MG-ADL score and log time, are presented in Supplemental Figure 5.

Results from the sensitivity (tipping point) analysis conducted to evaluate the choice of percentage by which to down-weight the priors (from 0% to 100%) confirmed that the results of the MIA were not impacted by the choice of this weight (data not shown). In sensitivity analysis 2 the use of a non-informative (vague) prior, corresponding to a 100% down-weighting of the external data, showed similar results to the primary analysis. The difference in predicted mean CFB in MG-ADL score between the zilucoplan and control groups was −2.51 (95% CrI: −3.64, −1.36) at week 24.

**Figure 3.**
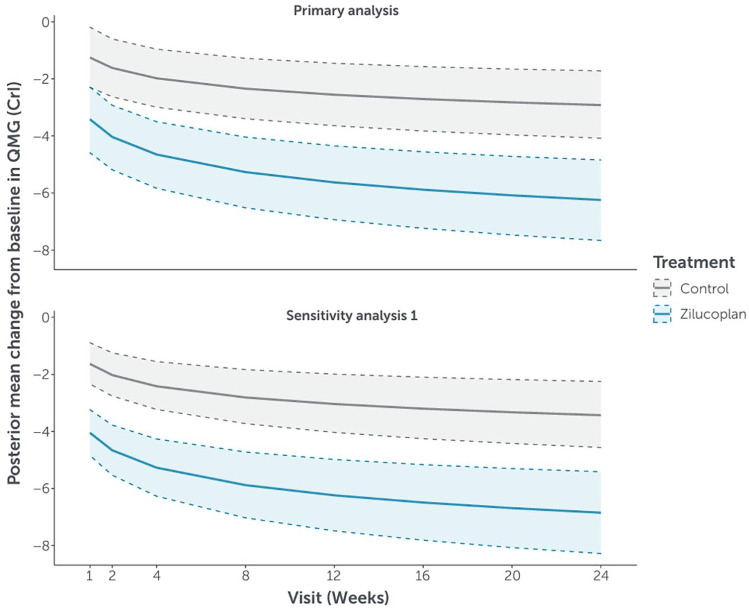
Evolution of predicted mean change from baseline (and associated 95% CrI) in QMG score through week 24. Primary analysis: Data from the double-blind studies, including RAISE-XT data for patients randomised to zilucoplan in the double-blind studies, were included in Part 2. A linear model with log time using the historical prior. Sensitivity analysis 1: Placebo data from the phase II study were included in Part 1. Data from RAISE only were included in Part 2. A linear model with log time using the historical prior. CrI, credible interval; QMG, quantitative myasthenia gravis.

The evolution of predicted mean CFB over time up to week 24 in QMG score for the zilucoplan and control groups was consistent with that observed in MG-ADL score (Figure 3 and Table 2).

No overlap was observed between the 95% CrIs of the predicted mean CFB for the zilucoplan and control groups. The distribution of the difference between the zilucoplan and control groups in the predicted mean CFB to week 24 in QMG score is not shown. When assessing QMG score, the posterior probability of an improved treatment effect with zilucoplan versus control as measured by the mean CFB at week 24 was >99.9%. There was a 67.5% probability that the difference in the predicted mean CFB in QMG score at week 24 between zilucoplan and the control group was greater than the clinically meaningful threshold (⩾3.0-point reduction).

## Discussion

The results of this MIA, which used data extracted from three types of external sources, are consistent with the 12-week effect observed with zilucoplan in phase II and phase III clinical studies and demonstrate the maintenance of efficacy of zilucoplan versus control up to 24 weeks. The application of an MIA to extend the duration of a placebo-controlled treatment period through the incorporation of historical data is novel, and this is the first time that such an analysis has been applied in gMG. Results of the sensitivity analyses reported, and all other pre-specified sensitivity analyses conducted for the MIA that are not shown here, corroborated those observed in the primary analysis, translating robustness to the likelihood of zilucoplan clinical efficacy up to 24 weeks. This MIA demonstrated that the posterior probability of zilucoplan having a higher clinical benefit than the control group at week 24 was very high (*p* < 0.0001). This sustained efficacy of zilucoplan has also been demonstrated in the ongoing RAISE-XT study following 24 weeks of zilucoplan treatment and beyond,^11^ confirming the reliability of the results of this MIA.

At week 12, the difference in predicted mean CFB in MG-ADL score between the zilucoplan and control groups (Bayesian MIA; mean difference vs control: −2.27) was of a similar magnitude to the results reported at week 12 in the RAISE study (frequentist approach using an MMRM model; LS mean difference vs placebo: −2.09).^9^ This slight difference observed at week 12 is not clinically meaningful and is likely due to the variance in approach (Bayesian using historical prior vs frequentist), confirming the reliability of the MIA. Similarly, modelled data for QMG score were consistent with those observed for MG-ADL score and with the results obtained in the RAISE study.^9^ In addition, the sensitivity analysis conducted using the mITT_RAISE_ population, which only utilised data from the pivotal RAISE study in Part 2, confirmed the robustness of the MIA by allowing for the comparison of posterior means at week 12 from the MIA to the week 12 RAISE study results. In this sensitivity analysis, the phase II study was included in the control meta-regression (Part 1) to further strengthen the informative prior. The consistency in results between the primary and sensitivity analysis confirms the reliable maintenance of efficacy of zilucoplan compared with a control for up to 24 weeks. Of note, while minimal overlap was observed between the 95% CrIs of the predicted mean CFB in MG-ADL score at week 24 for the zilucoplan and control groups in the primary analysis, the posterior probability that there is a worsened or similar effect with zilucoplan versus the control is <0.0001. Further, no such overlap was observed in the sensitivity analysis. In addition, these modelled data show no sign of waning efficacy with zilucoplan through weeks 12−24, supporting the long-term efficacy of zilucoplan in patients with gMG.

Furthermore, results of the sensitivity analysis performed on the mITT_RAISE_ population for predicted mean CFB to week 24 in MG-ADL score for the zilucoplan group are comparable to those observed in a post hoc analysis of RAISE-XT. The post hoc analysis only included patients from the RAISE study, with rescue therapy and discontinuations imputed as treatment failure, and death imputed as the worst possible score, providing additional evidence of the reliability of the MIA. Data from the ongoing OLE study, RAISE-XT, further elucidate the maintenance of efficacy effect beyond 24 weeks and are consistent with these modelling data.^11^

The robustness of our study is evident in the consistency of data across analyses. An additional strength of our methodology is that the Bayesian statistical approach provides explicit assumptions about the probability of an improved treatment effect with zilucoplan versus the control. By providing an estimated distribution of the observed efficacy, Bayesian approaches more closely model clinical behaviour.^16^ Further, a Bayesian approach also has the potential to reduce the number of patients required for a study, and hence, result in fewer concurrent control patients, without reducing the study’s power to detect an effect.^16^ Through modelling control data up to week 24, this MIA limited patients’ exposure to placebo in the phase III RAISE study to 12 weeks, preventing the unnecessary withholding of treatment for a further 12 weeks. This methodology could be used to inform the length of future studies or reduce the number of concurrent control patients required.

There are some limitations, however, including the identification of several potential biases. First, the linear model (with log time) assumes that disease progression is similar in both treatment groups, serving as a potential, though unlikely, violation of assumptions. An Emax model was employed in a supplementary analysis to ensure that the log-linear model did not bias the posterior distribution; results were consistent with the primary analysis. Second, estimation of the response to control beyond 12 weeks and up to 24 weeks may have been subject to selection and publication bias. The IPTW could only be used on two data sources as individual patient-level data were not available for data sources from the SLR. In addition, it is acknowledged that any relevant clinical study data published outside of the search period within which the SLR was conducted will not have been captured. However, the subsequent inclusion of ravulizumab data that were published following completion of the SLR mitigates this. Third, reporting of MG-ADL scores in the Swedish MG Registry was quite low, which led to a small sample of patients being selected from this data source (only 16 participants). This limitation stems directly from the challenges that arise with the use of real-world data, which are often incomplete, heterogeneous and subject to various measurement errors.^31^ Lastly, the use of OLE data for zilucoplan between week 12 and week 24 may be subject to bias due to the switch from double-blind to open-label. However, this bias is expected to be limited since the model was adjusted for the study period (double-blind vs open-label). The wide range of sensitivity analyses with consistent results (data not shown) support the belief that the impact of these limitations is likely to be minor.

## Conclusion

This MIA estimated the maintenance of efficacy of zilucoplan versus an external control group for up to 24 weeks in lieu of placebo-controlled data. The consistency and robustness of the results from the primary and sensitivity analyses of this MIA support the hypothesis that the observed statistically significant treatment effect of zilucoplan on MG-ADL and QMG scores at week 12, as seen in the double-blind, placebo-controlled, phase III RAISE study, is sustained through to 24 weeks.

## Supplemental Material

sj-docx-1-tan-10.1177_17562864241279125 – Supplemental material for Maintenance of zilucoplan efficacy in patients with generalised myasthenia gravis up to 24 weeks: a model-informed analysis Supplemental material, sj-docx-1-tan-10.1177_17562864241279125 for Maintenance of zilucoplan efficacy in patients with generalised myasthenia gravis up to 24 weeks: a model-informed analysis by Guillemette de la Borderie, Damien Chimits, Babak Boroojerdi, Melissa Brock, Petra W. Duda, Fiona Grimson, Paul Mahoney, Foteini Strimenopoulou, Gary Cutter, Inmaculada Aban, Susanna Brauner, Malin Petersson, James F. Howard and Nathan Bennett in Therapeutic Advances in Neurological Disorders

## Declarations

**Ethics approval and consent to participate:** The analysis of data from the Swedish MG Registry was approved by the Regional Ethics Review Board of Stockholm, Sweden (Dnr 2016/828-31), allowing for retrospective analysis of data collected in the registry. An institutional review board or independent ethics committee for each participating site for the phase II study, RAISE study and RAISE-XT study approved the protocols. All participants provided written informed consent. The approving institutional review boards and independent ethics committees for the RAISE-XT study have been published. Each participating site for the MGTX study received approval from a local institutional review board or ethics committee, and each patient provided written informed consent before enrollment. The MGTX data were requested through the National Institute of Neurological Disorders and Stroke, which is part of the United States National Institutes of Health. The MGTX study was required by the terms of its funding to make the data public.

**Consent for publication:** Not applicable.

**Competing interests:** Guillemette de la Borderie, Babak Boroojerdi, Melissa Brock, Petra W. Duda, Fiona Grimson, Paul Mahoney, Foteini Strimenopoulou and Nathan Bennett are employees and shareholders of UCB. Damien Chimits was an employee and shareholder of UCB and now works for argenx. Gary Cutter has served on data and safety monitoring boards for Applied Therapeutics, AI therapeutics, AMO Pharma, AstraZeneca, AveXis Pharmaceuticals, Bristol Myers Squibb (Celgene), CSL Behring, Cynata Therapeutics, DiaMedica Therapeutics, Horizon Pharmaceuticals, Immunic, Karuna Therapeutics, Kezar Life Sciences, Mapi Pharma, Merck, Mitsubishi Tanabe Pharma, Opko Biologics, Prothena Biosciences, Novartis, Pipeline Therapeutics, Regeneron, Sanofi-Aventis, Reata Pharmaceuticals, Teva Pharmaceuticals, United BioSource LLC, University of Texas Southwestern, University of Pennsylvania, Visioneering Technologies, Inc. He has been a Consultant or served on advisory boards for Alexion, Antisense Therapeutics, Avotres, Biogen, Clene Nanomedicine, Clinical Trial Solutions LLC, Endra Life Sciences, Cognito Therapeutics, Entelexo Biotherapeutics, Genzyme, Genentech, GW Pharmaceuticals, Hoya Corporation, Immunic, Immunosis, Klein-Buendel Incorporated, Linical, Merck Serono, Novartis, Perception Neurosciences, Protalix Biotherapeutics, Regeneron, Roche, SAB Biotherapeutics and Sapience Therapeutics. Dr Cutter is employed by the University of Alabama at Birmingham and President of Pythagoras, a private consulting company located in Birmingham, Alabama. Inmaculada Aban is a Consultant for Roche and has received research support from Alexion Pharmaceuticals, argenx, the Myasthenia Gravis Foundation of America, Ra Pharmaceuticals (now UCB) and Verona. Susanna Brauner has received research grants from The Stockholm Healthcare Region, The Swedish Medical Society, UCB and Janssen Pharmaceuticals. Malin Petersson is a PhD student supported by an unrestricted research grant to Fredrik Piehl from UCB. James F. Howard Jr has received research support (paid to his institution) from Ad Scientiam, Alexion AstraZeneca Rare Disease, argenx, Cartesian Therapeutics, the Centers for Disease Control and Prevention (Atlanta, GA, USA), the Myasthenia Gravis Foundation of America, the Muscular Dystrophy Association, the National Institutes of Health (including the National Institute of Neurological Disorders and Stroke and the National Institute of Arthritis and Musculoskeletal and Skin Diseases), NMD Pharma, PCORI and UCB; he has received honoraria/consulting fees from AcademicCME, Alexion AstraZeneca Rare Disease, argenx, Biohaven Ltd, Biologix Pharma, CheckRare CME, F. Hoffmann-LaRoche, Horizon Therapeutics (now Amgen), Medscape CME, Merck EMD Serono, NMD Pharma, Novartis, PeerView CME, Physicians’ Education Resource (PER) CME, PlatformQ CME, UCB, Regeneron Pharmaceuticals, Sanofi US and Zai Labs; and has received non-financial support from Alexion AstraZeneca Rare Disease, argenx, Toleranzia AB and UCB.

**Availability of data and materials:** Underlying data from this manuscript may be requested by qualified researchers 6 months after product approval in the United States and/or Europe, or global development is discontinued, and 18 months after trial completion. Prior to use of the data, proposals need to be approved by an independent review panel at https://www.Vivli.org and a signed data-sharing agreement will need to be executed. All documents are available in English only, for a pre-specified time, typically 12 months, on a password-protected portal. Data from the Swedish MG Registry are available at reasonable request at anonymised and aggregated level.
