# Supplementary material for: Maintenance of zilucoplan efficacy in patients with generalised myasthenia gravis up to 24 weeks: a model-informed analysis
Source: Ther Adv Neurol Disord. 2024 Sep 21;17:17562864241279125. doi: 10.1177/17562864241279125 (PMC11418339; doi:10.1177/17562864241279125)
Supplement: sj-docx-1-tan-10.1177_17562864241279125 – Supplemental material for Maintenance of zilucoplan efficacy in patients with generalised myasthenia gravis up to 24 weeks: a model-informed analysis [file sj-docx-1-tan-10.1177_17562864241279125.docx]

# Maintenance of zilucoplan efficacy in patients with generalised myasthenia gravis up to 24 weeks: A model-informed analysis

**Guillemette de la Borderie, MSc^1^, Damien Chimits, MSc^1^, Babak Boroojerdi, MD^2^, Melissa ‍Brock, PharmD^3^, Petra W. Duda, MD, PhD^4^, Fiona Grimson, PhD^5^, Paul Mahoney, PhD^5^, Foteini Strimenopoulou, PhD^5^, Gary Cutter, PhD^6^, Inmaculada Aban, PhD^6^, Susanna Brauner, MD, PhD^7,8^, Malin Petersson, MD^7^,
James F. Howard Jr., MD^9^, Nathan Bennett, PhD^3^**

^1^UCB Pharma, Colombes, France; ^2^UCB Pharma, Monheim, Germany; ^3^UCB Pharma, Morrisville, NC, USA; ^4^UCB Pharma, Cambridge, MA, USA; ^5^UCB Pharma, Slough, UK; ^6^Department of Biostatistics, The University of Alabama at Birmingham School of Public Health, Birmingham, AL, USA; ^7^Department of Clinical Neuroscience, Karolinska Institutet, Stockholm, Sweden; ^8^Department of Neurology, Karolinska University Hospital, Stockholm, Sweden; ^9^Department of Neurology, The University of North Carolina at Chapel Hill, Chapel Hill, NC, USA

Table of contents

[Supplementary Material 3](#_Toc169685783)

[Supplementary Table 1. Search terms used in the SLR 3](#_Toc169685784)

[Supplementary Table 2. Data used in the control meta-regression (Part 1) 5](#_Toc169685785)

[Supplementary Table 3. Data used in the individual patient-level data combined analysis (Part 2) 8](#_Toc169685786)

[Supplementary Figure 1. Flow chart of studies selected in the SLR 9](#_Toc169685787)

[Supplementary Figure 2. Predicted mean change from baseline in MG-ADL score for the control group of the MIA and external control data (control meta-regression) 10](#_Toc169685788)

[Supplementary Figure 3. Histogram of the predicted mean change from baseline distribution at Week 24 in MG-ADL score by treatment group 11](#_Toc169685789)

[Supplementary Figure 4. Evolution of predicted mean change from baseline (and associated 95% CrI) and frequentist inference in MG-ADL score through Week 24 12](#_Toc169685790)

[Supplementary Figure 5. Supplementary analysis using an Emax model for the evolution of predicted mean change from baseline (and associated 95% CrI) in MG-ADL score through Week 24 13](#_Toc169685791)

[Supplementary Appendix 1 14](#_Toc169685792)

[Statistical methodology: A Bayesian model-informed analysis (MIA) using a
two-stage approach 14](#_Toc169685793)

[Part 1: Control meta-regression (including placebo and standard of care) 15](#_Toc169685794)

[Part 2: Individual patient-level data combined analysis 16](#_Toc169685795)

[References 17](#_Toc169685796)

# Supplementary Material

## Supplementary Table 1. Search terms used in the SLR

|  | **Query** | **Facet** | **Results** |
| --- | --- | --- | --- |
|  | **Embase^®^ and MEDLINE^®^ (via Embase.com on 27 January 2022)** | | |
| #1 | 'myasthenia gravis'/syn OR 'myasthenia' OR myastheni* OR (('acetylcholine receptor antibody' OR 'achr' OR 'muscle specific kinase antibody' OR 'lipoprotein related protein 4' OR 'lrpr4' OR 'seronegative') NEAR/4 ('myasthen*' OR 'myasthenia gravis')) | Disease | 32,702 |
| #2 | clinical trial'/exp OR 'randomization'/de OR 'controlled study'/de OR 'comparative study'/de OR 'single blind procedure'/de OR 'double blind procedure'/de OR 'crossover procedure'/de OR 'placebo'/de OR 'clinical trial' OR 'clinical trials' OR 'controlled clinical trial' OR 'controlled clinical trials' OR 'randomised controlled trial' OR 'randomized controlled trial' OR 'randomised  controlled trials' OR 'randomized controlled trials' OR 'randomisation' OR 'randomization' OR rct OR 'random allocation' OR 'randomly allocated' OR 'allocated randomly' OR placebo* OR 'prospective study'/de OR allocated NEAR/2 random OR random* NEAR/1 assign* OR random* OR (single OR double OR triple OR treble) NEAR/1 (blind* OR mask*) NOT ('case study'/de OR 'case report' OR 'abstract report'/de OR 'letter'/de) | Study design | 11,189,372 |
| #3 | cohort analysis'/exp OR 'longitudinal study'/exp OR 'prospective study'/exp OR 'follow up'/exp OR 'major clinical study'/exp OR 'clinical trial'/exp OR 'clinical article'/exp OR 'intervention study'/exp OR 'survival'/exp OR cohort*:ab,ti OR ((('follow up'  OR follow-up) NEXT/1 (study OR studies)):ab,ti) OR ((clinical NEXT/1 trial*):ab,ti) OR 'retrospective study'/exp OR 'case control study'/exp OR ((case* NEXT/1 control*):ab,ti) | Study design | 10,396,238 |
| #4 | #2 OR #3 | Study design | 16,029,298 |
| #5 | #1 AND #4 | Combined facet | 14,662 |
| #6 | #5 AND ([conference review]/lim OR [editorial]/lim OR [letter]/lim OR [note]/lim OR [review]/lim OR [short survey]/lim) | Animal studies/editorials/ reviews | 1972 |
| #7 | #5 AND [animals]/lim NOT ([humans]/lim AND [animals]/lim) | Animal studies/editorials/ reviews | 817 |
| #8 | #6 OR #7 | Final hits | 2764 |
| #9 | #5 NOT #8 | Final hits | **11,898** |
| #10 | #9 AND [English]/lim | Final hits | **10,580** |
|  | **Cochrane (via Cochrane.com on 27 January 2022)** | | |
| #1 | MeSH descriptor: [myasthenia gravis] explode all trees | Disease | 222 |
| #2 | “myasthenia” OR “myastheni*” | Disease | 783 |
| #3 | (“acetylcholine receptor antibody” OR “achr” OR “muscle specific kinase antibody” OR “lipoprotein related protein 4” OR “lrpr4” OR “seronegative”) NEAR/4 (“myasthen*” OR “myasthenia gravis”) | Disease | 49 |
| #4 | #1 OR #2 OR #3 | Disease | 787 |
| #5 | #4 in Trials | Final hits | **357** |
|  | **MEDLINE^®^ In-process (via PubMed.com on 27 January 2022)** | | |
| #1 | “myasthenia gravis” | Disease | 19,120 |
| #2 | #1 AND (inprocess[sb] OR pubstatusaheadofprint) | Final hits | **517** |

SLR, systematic literature review.

## Supplementary Table 2. Data used in the control meta-regression (Part 1)

| Type of data/source | Description | | Selection criteria |
| --- | --- | --- | --- |
| Aggregate data /SLR | BEL115123, belimumab  (Phase 2; NCT01480596)^1^ | Randomised study of adjunctive belimumab in patients with generalised myasthenia gravis | Any placebo-controlled randomised clinical studies in patients aged ≥18 years with gMG (moderate to severe MG) and at least one MG-ADL assessment reported up to Week 28 |
|  | REGAIN, eculizumab  (Phase 3; NCT01997229)^2^ | Safety and efficacy of eculizumab in anti-acetylcholine receptor antibody-positive refractory generalised myasthenia gravis (REGAIN): a Phase 3, randomised, double-blind, placebo-controlled, multicentre study |  |
|  | Tacrolimus (Phase 3; NCT01325571)^3^ | Tacrolimus in the treatment of myasthenia gravis in patients with an inadequate response to glucocorticoid therapy: randomised, double-blind, placebo-controlled study conducted in China |  |
|  | Ravulizumab  (Phase 3; NCT03920293)^4^ | Terminal complement inhibitor ravulizumab in generalised myasthenia gravis |  |
|  | ADAPT, efgartigimod (Phase 3; NCT03669588)^5^ | Safety, efficacy and tolerability of efgartigimod in patients with generalised myasthenia gravis (ADAPT): a multicentre, randomised, placebo-controlled, Phase 3 trial |  |
|  | Rozanolixizumab  (Phase 2; NCT03052751)^6^ | Efficacy and safety of rozanolixizumab in moderate-to-severe generalised myasthenia gravis: a Phase 2a randomised control trial |  |
| Individual patient-level data/Swedish MG Registry | Aggregate data obtained from a MMRM on a selected cohort of patients comparable to RAISE patients after odds weighting. | | Patients aged ≥18 years with at least one MG-ADL assessment up to 28 weeks and MG-ADL ≥6 at index (where the index date was defined as the first MG-ADL measurement ≥6) with:   - - a diagnosis of gMG   - a positive serology for AChR binding autoantibodies   - no thymectomy within six months prior to index date (achieved by censoring any data within the six months of the thymectomy procedure)   - no treatment with IVIg, SC immunoglobulin or PLEX within four weeks prior to index date (achieved by censoring this time period if these treatments were taken)   For QMG, the same selection criteria were used, replacing the threshold of MG-ADL ≥6 at index with QMG ≥12 at index (where the index date was defined as the first QMG measurement ≥12) |
| Individual patient-level data/MGTX study^7^ | Aggregate data obtained from a MMRM on a selected cohort of patients comparable to patients from the Phase 3 RAISE study after odds weighting.  Censoring was employed by the model to better reflect the exclusion criteria of the Phase 3 RAISE study. The prednisone monotherapy group was censored for the first three months to remove the period where the largest number of dose changes in steroids occurred. The thymectomy treatment group was censored for the first 12 months to remove the effect of thymectomy. The two treatment groups were then naïvely pooled. | | Patients aged ≥18 years with MG-ADL ≥6 at index (where the index date was defined as the first MG-‍ADL measurement ≥6) and:   - - a diagnosis of gMG   - a positive serology for AChR binding autoantibodies   For QMG, the same selection criteria were used, replacing the threshold of MG-ADL ≥6 at index with QMG ≥12 at index (where the index date was defined as the first QMG measurement ≥12) |

AChR, acetylcholine receptor; gMG, generalised myasthenia gravis; IVIg, intravenous immunoglobulin; MG-ADL, Myasthenia Gravis Activities of Daily Living; MMRM; mixed model for repeated measurements; PLEX, plasma exchange; QMG, Quantitative Myasthenia Gravis; SC, subcutaneous; SLR, systematic literature review.

## Supplementary Table 3. Data used in the individual patient-level data combined analysis (Part 2)

| **Type of data** | **Source** | | **Description** | **Analysis population included in the primary/sensitivity analysis 1** |
| --- | --- | --- | --- | --- |
| Individual patient-level data | MG0009 (Phase 2; NCT03315130)^8^ | Phase 2 placebo and zilucoplan treatment group | An individual patient-level data combined analysis where the three clinical studies use the same primary estimand (i.e., intercurrent event handling strategy) as the Phase 3 RAISE study of zilucoplan^9^ | mITT (primary analysis) |
|  | RAISE (Phase 3; NCT04115293)^9^ | Phase 3 placebo and zilucoplan treatment group |  | Mitt (primary analysis)/ mITT_RAISE_ (sensitivity analysis 1) |
|  | RAISE-XT (OLE; NCT04225871)^10^ | Only included for patients randomised to zilucoplan in the Phase 2 or RAISE studies |  | mITT (primary analysis) |

mITT, modified intention-to-treat; OLE, open-label extension.

## Supplementary Figure 1. Flow chart of studies selected in the SLR


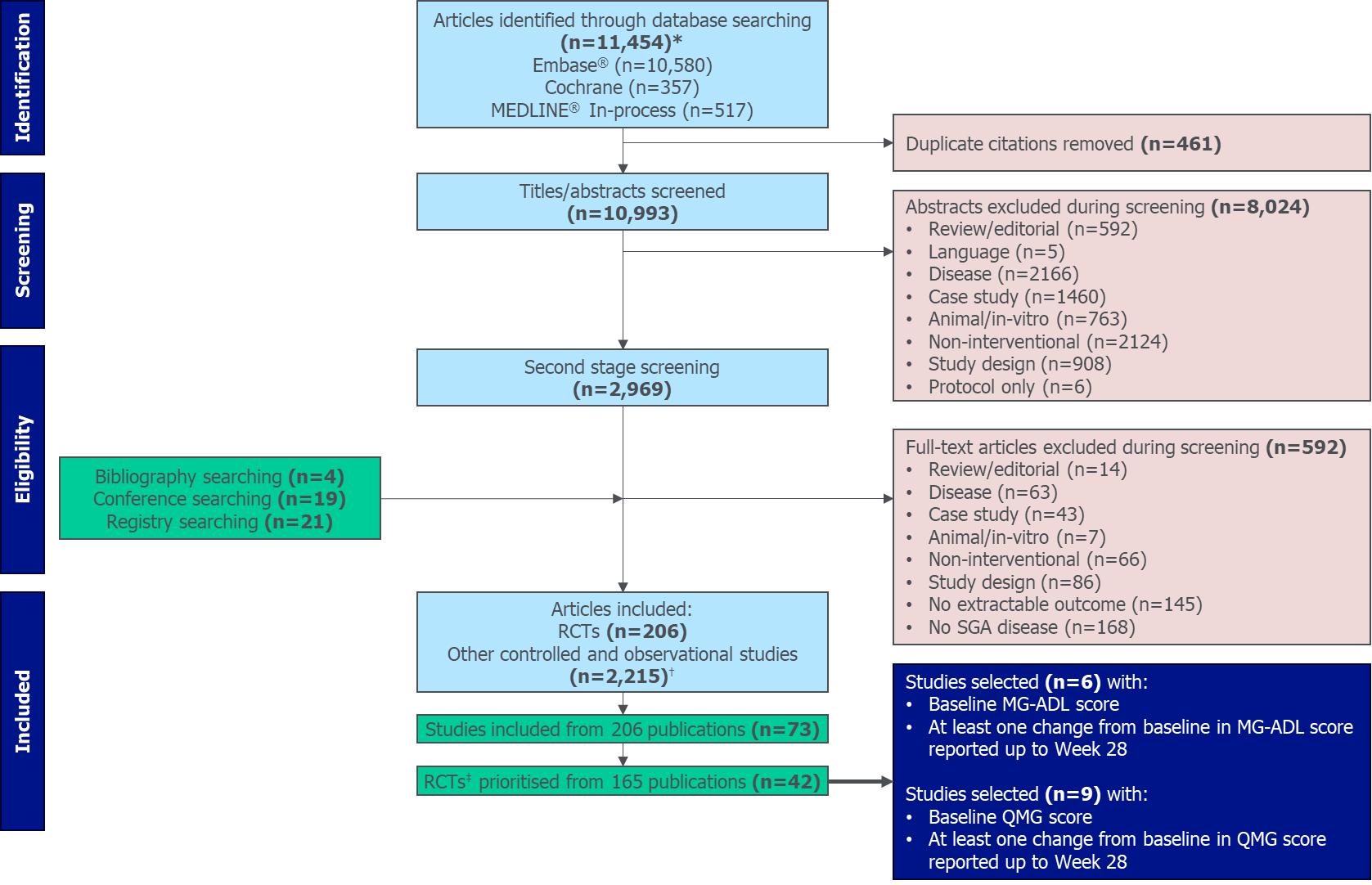


*Search terms are listed in **Supplementary Table 1**. ^†^Includes publications that were marked as observational during the first stage screening based on titles and abstracts. ^‡^Represent only the gMG population (mild to moderate, mild to severe, moderate to severe, severe, refractory and exacerbating groups).
MG-ADL, Myasthenia Gravis Activities of Daily Living; QMG, Quantitative Myasthenia Gravis; RCT, randomised controlled trial; SGA, small for gestational age; SLR, systematic literature review.

## Supplementary Figure 2. Predicted mean change from baseline in MG-ADL score for the control group of the MIA and external control data (control meta-regression)


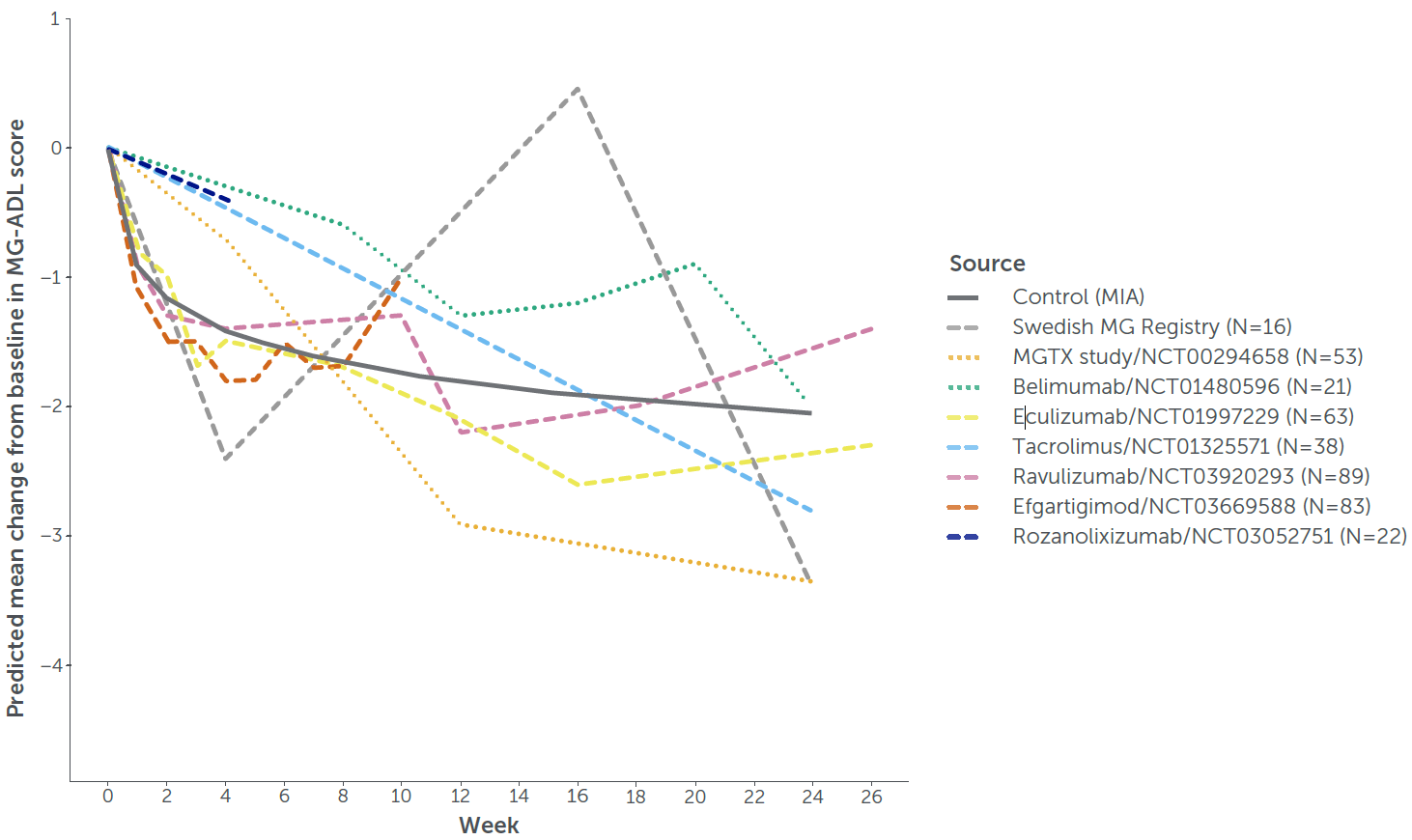


External data after odds weighting (N=385).
MG-ADL, Myasthenia Gravis Activities of Daily Living; MIA, model-informed analysis.

## Supplementary Figure 3. Histogram of the predicted mean change from baseline distribution at Week 24 in MG-ADL score by treatment group


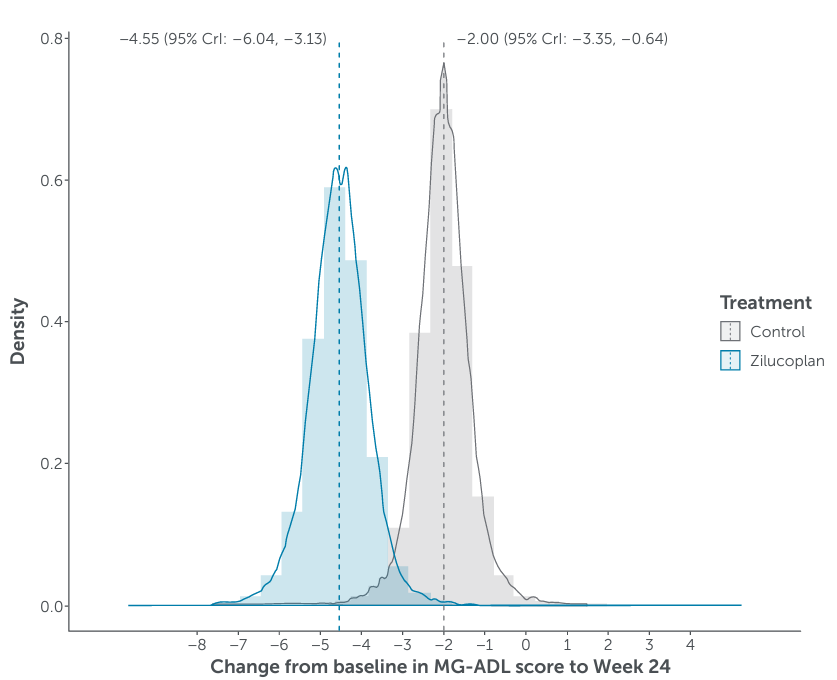


Primary analysis: Data from the double-blind studies, including RAISE-XT data for patients randomised to zilucoplan in the double-blind studies, were included in Part 2. A linear model with log time using the historical prior.
MG-ADL, Myasthenia Gravis Activities of Daily Living.

## Supplementary Figure 4. Evolution of predicted mean change from baseline (and associated 95% CrI) and frequentist inference in MG-ADL score through Week 24


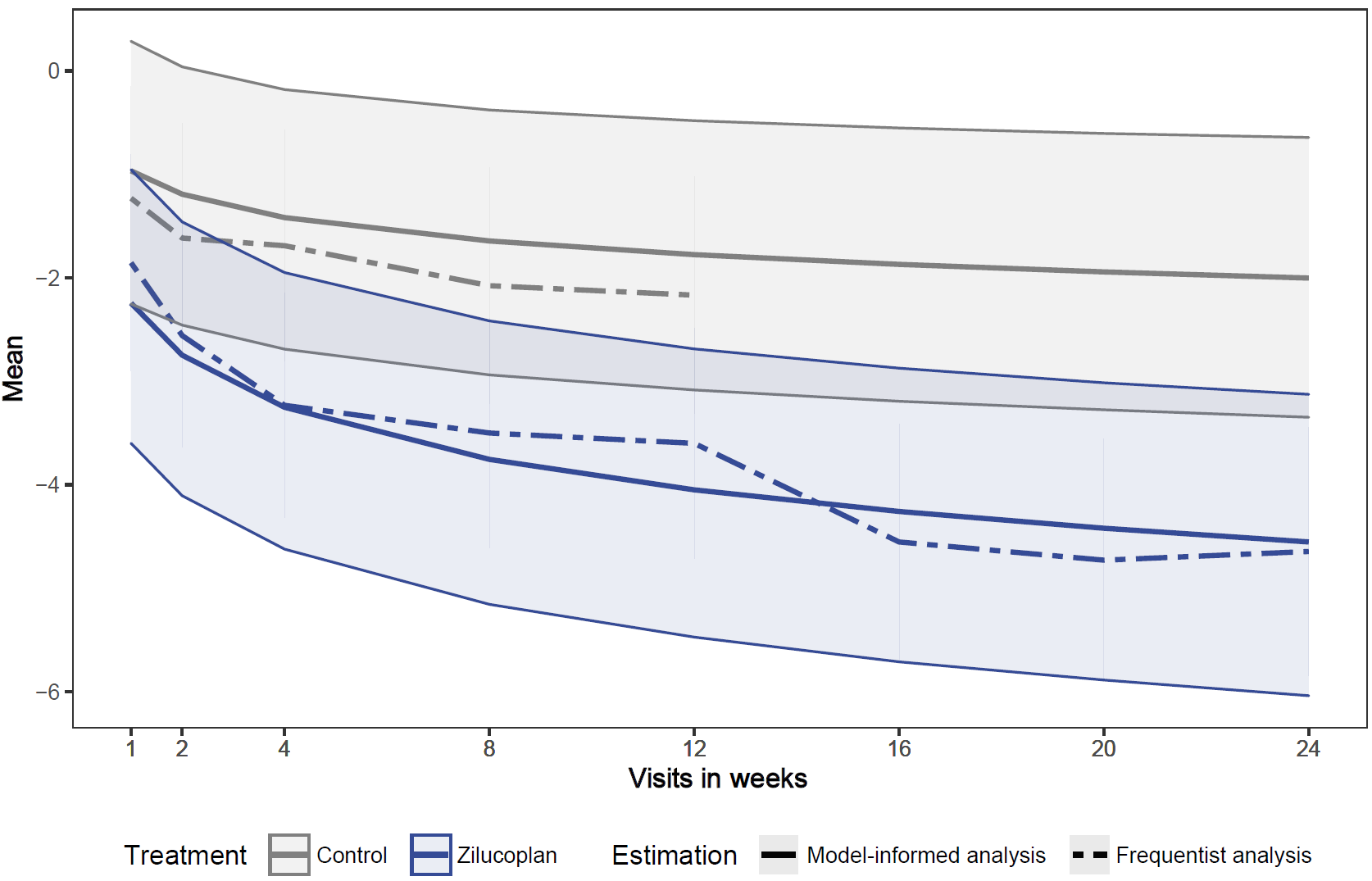


Primary analysis (MIA): Data from the double-blind studies, including RAISE-XT data for patients randomised to zilucoplan in the double-blind studies, were included in Part 2. A linear model with log time using the historical prior.

Frequentist inference: Mixed model of repeated measures adjusted on study, treatment, MG-ADL and QMG score at baseline, region, visit, baseline by visit and treatment by visit.

CrI, credible interval; MG-ADL, Myasthenia Gravis Activities of Daily Living, MIA, model-informed analysis; QMG, Quantitative Myasthenia Gravis.

## Supplementary Figure 5. Supplementary analysis using an Emax model for the evolution of predicted mean change from baseline (and associated 95% CrI) in MG-ADL score through Week 24


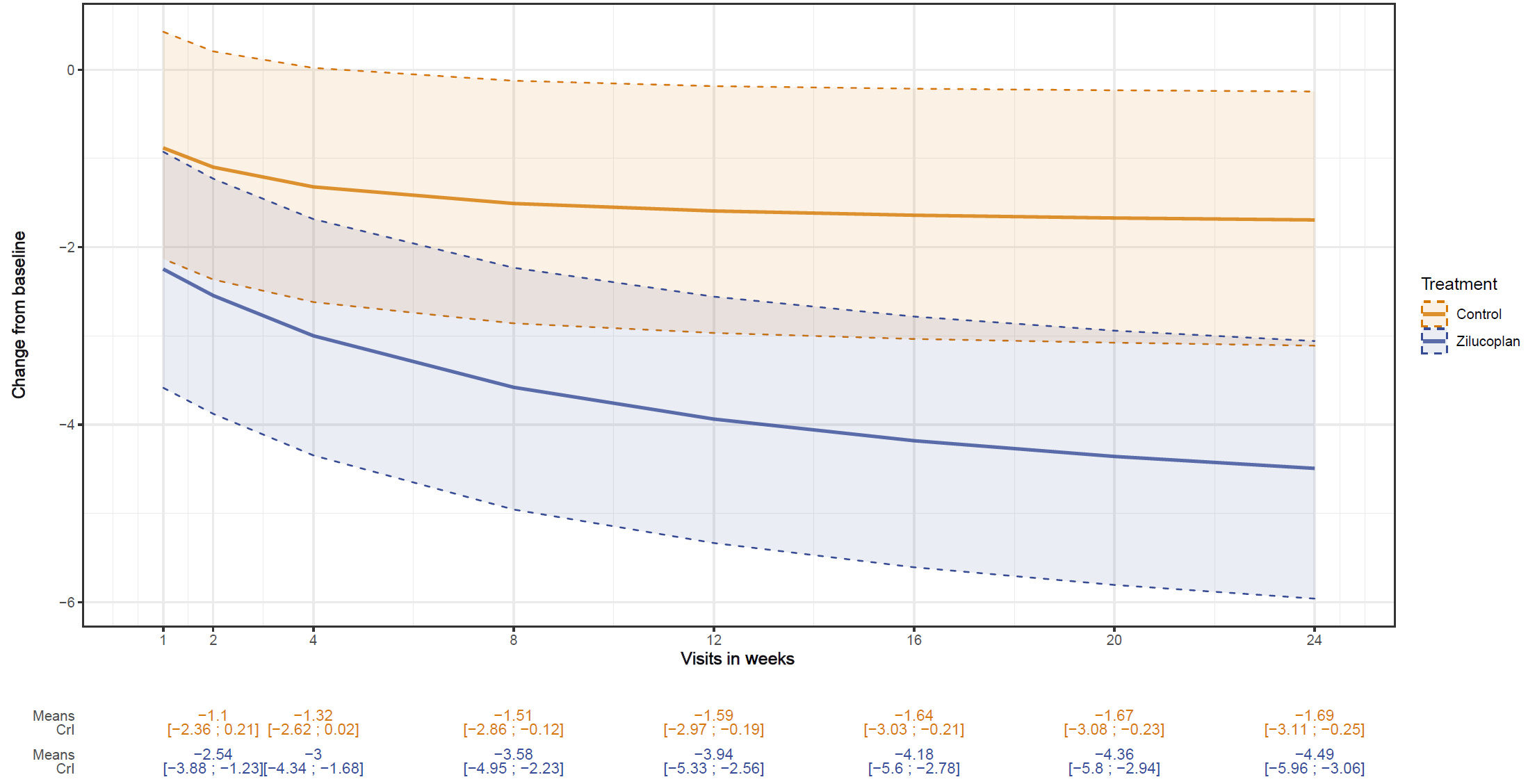


Supplementary analysis: Data from the double-blind studies, including RAISE-XT data for patients randomised to zilucoplan in the double-blind studies, were included in Part 2. An Emax model with continuous time using the historical prior.

CrI, credible interval; MG-ADL, Myasthenia Gravis Activities of Daily Living.

# Supplementary Appendix 1

## Statistical methodology: A Bayesian model-informed analysis (MIA) using a two-stage approach

This combined-studies analysis consisted of two parts; the meta-regression on control summary data to build an informative prior (i.e., historical prior) and the combined-individual patient-level analysis informed by the results of Part 1. The primary analysis of both parts used a linear model with log time. This model assumed that the disease progression was similar over time in the zilucoplan and control groups. The main outcome of this analysis was the prediction of the treatment effect of zilucoplan versus the control at Week 24. The models were run in the JAGS software on R version 3.6.2.


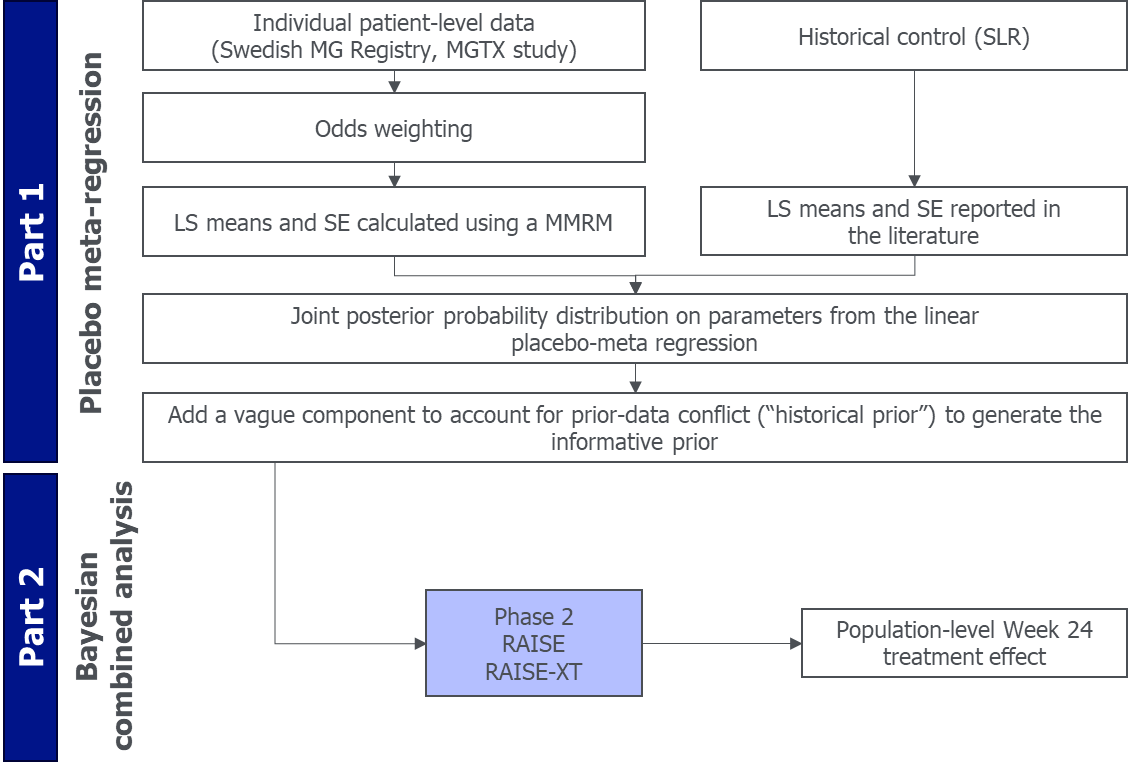


LS, least square; MMRM, mixed model with repeated measurements; SE, standard error; SLR, systematic literature review.

### Part 1: Control meta-regression (including placebo and standard of care)

Part 1 synthesised information based on the summary data for response to the control over time through a meta-regression model using aggregate data. The outcome of this Bayesian meta-regression on aggregate data was the posterior distribution of the model parameters: the overall mean response (intercept), the slope of the response over time, the study-to-study variation and the baseline effect, used as an informative prior in Part 2.

The following linear mixed model for the Myasthenia Gravis Activities of Daily Living (MG-ADL) change from baseline means ($y_{sj}$) in the historical study *s* at time point *j* was assumed using the centered log time and centered baseline MG-ADL mean as covariates:

$$y_{sj}\sim N\left( \alpha_{s}+\gamma*(log {time}_{sj}-m_{\log time})+\beta\left( B_{s}{-m}_{B} \right), \sigma_{sj}^{2} \right)$$

${time}_{sj}$ was the $j$^th^ timepoint (in weeks) in the $s$^th^ study. $\sigma_{sj}$ was the standard error of the mean change from baseline at the $j$^th^ timepoint in the $s$^th^ study, assumed to be known. $B_{s}$ was the baseline MG-ADL mean in the $s$^th^ study. $m_{\log time}$ and $m_{B}$ were the mean log time and the mean baseline across all studies, respectively.

The coefficients of the centered time and centered baseline variables, $\gamma$ and $\beta$, respectively, were assumed to be the same between studies. The intercept, $\alpha_{s}$, which was allowed to differ between studies, was normally distributed with population mean equal to $\alpha$ and study-to-study standard deviation $(\tau)$.

This study-to-study standard deviation $\tau$ was set to have a weakly informative prior^11,12^ using the following distribution, where $\sigma$ was the sampling standard deviation estimated by the available data:

$$\tau\sim HN(0,\sigma^{2}/4)$$

The priors for the rest of the parameters in the model were vague.

The outcome of this analysis was samples from the posterior distribution of each parameter in the model. To use these outcomes as prior distributions to Part 2 of the model, a parametric distribution was fitted to the samples. A flexible family of distributions was used: a normal or multivariate normal mixture, depending on the correlation between the parameters.^13^ A gamma distribution was used for $\tau$. For each parameter, the mixture providing the lower Akaike’s information criterion (AIC) value was then selected.

A key assumption for the validity of this analysis was that all external data were relevant to the RAISE study and could be assumed exchangeable. This was achieved through careful selection of the studies during the systematic literature review. However, the assumption of exchangeability might not hold due to differing inclusion and exclusion criteria to those used in the RAISE study. To address these issues, a robust approach was proposed by adding a vague component^14^ to the resulting posterior distributions to get a rapid adaptation to prior-data conﬂict, down-weighted with a 30% probability that the true model parameters in RAISE were not similar to the parameters estimated. Down-weighting of the prior information was allowed in a dynamic manner in the analysis of Part 2. This means that the more discrepant the information in the Phase 2 and RAISE studies was to the priors, the more the historical prior would be dynamically down-weighted.

### Part 2: Individual patient-level data combined analysis

Once the historical control prior was obtained (Part 1), a combined analysis of the two zilucoplan studies (Phase 2 and RAISE) was performed using this informative historical prior. Both studies had double-blind data up to Week 12. Additionally, data up to Week 24 from the extension portion of the Phase 2 study and the open-label extension study RAISE-XT were included as observed data for patients randomised to zilucoplan in the Phase 2 study and the RAISE study, respectively.

The following linear mixed model for the MG-ADL change from baseline responses ($y_{ijs}$) in the Phase 2 and RAISE studies for study participant i at timepoint j in study s (Phase 2 or RAISE) was assumed using the centered log-time, centered baseline MG-ADL, treatment, and interaction of log time with treatment as terms in the model:

$$y_{ijs}\sim N\left( \alpha_{is}+\gamma_{i}*(log {time}_{sj}-m_{\log time})+\beta*{(B}_{is}-m_{B})+\delta*{trt}_{is}+\omega{*trt}_{is}*(log {time}_{sj}-m_{\log time})+\lambda*t_{ij},\sigma^{2} \right)$$

The open-label indicator $t_{ij}$ was equal to 0 if the timepoint j of subject $i$ was in the double-blind portion and 1 if the timepoint j of subject $i$ was in open-label portion. $\lambda$ was the shift parameter added to model the difference between the double-blind and the open-label portion

$\alpha_{is}$ was the intercept, which varies between patients and studies i.e., the mean change from baseline when all explanatory variables in the model were zero of patient $i$in study $s$, $\gamma_{i}$ was the coefficient of the centered log-time and it was allowed to vary between patients. $\beta,$ $\delta$ and $\omega$were the coefficients of the centered baseline, treatment (${trt}_{is})$ and centered time by treatment interaction terms, respectively.

$m_{\log time}$ and $m_{B}$ were the mean log time and the mean baseline across all studies in the meta-regression part, respectively.

The intercept term $\alpha_{is}$ and the time slope $\gamma_{i}$ were expected to be correlated as both varied by subject, therefore, it was assumed to come from a bi-variate normal distribution.

The parameters $\alpha$,$\beta, \gamma$ and $\tau$ had the informative prior described in Part 1. No prior knowledge was assumed to be available for the other parameters in the model.

# References

1. Hewett K, Sanders DB, Grove RA, et al. Randomized study of adjunctive belimumab in participants with generalized myasthenia gravis. *Neurology* 2018; 90: e1425–e1434.
2. Howard JF Jr., Utsugisawa K, Benatar M, et al. Safety and efficacy of eculizumab in anti-acetylcholine receptor antibody-positive refractory generalised myasthenia gravis (REGAIN): a Phase 3, randomised, double-blind, placebo-controlled, multicentre study. *Lancet Neurol* 2017; 16: 976–986.
3. Zhou L, Liu W, Li W, et al. Tacrolimus in the treatment of myasthenia gravis in patients with an inadequate response to glucocorticoid therapy: randomized, double-blind, placebo-controlled study conducted in China. *Ther Adv Neurol Disord* 2017; 10: 315–325.
4. Vu T, Meisel A, Mantegazza R, et al. Terminal complement inhibitor ravulizumab in generalized myasthenia gravis. *NEJM Evid* 2022; 1.
5. Howard JF Jr., Bril V, Vu T, et al. Safety, efficacy, and tolerability of efgartigimod in patients with generalised myasthenia gravis (ADAPT): a multicentre, randomised, placebo-controlled, Phase 3 trial. *Lancet Neurol* 2021; 20: 526–536.
6. Bril V, Benatar M, Andersen H, et al. Efficacy and safety of rozanolixizumab in moderate to severe generalized myasthenia gravis: a Phase 2 randomized control trial. *Neurology* 2021; 96: e853–e865.
7. Wolfe GI, Kaminski HJ, Aban IB, et al. Randomized trial of thymectomy in myasthenia gravis. *N Engl J Med* 2016; 375: 511–522.
8. Howard JF Jr., Nowak RJ, Wolfe GI, et al. Clinical effects of the self-administered subcutaneous complement inhibitor zilucoplan in patients with moderate to severe generalized myasthenia gravis: results of a Phase 2 randomized, double-blind, placebo-controlled, multicenter clinical trial. *JAMA Neurol* 2020; 77: 582–592.
9. Howard JF Jr., Bresch S, Genge A, et al. Safety and efficacy of zilucoplan in patients with generalised myasthenia gravis (RAISE): a randomised, double-blind, placebo-controlled, Phase 3 study. *Lancet Neurol* 2023; 22: 395–406.
10. Howard JF Jr, Bresch S, Farmakidis C, et al. Long-term safety and efficacy of zilucoplan in patients with generalized myasthenia gravis: interim analysis of the RAISE-XT open-label extension study. *Ther Adv Neurol Disord* 2024; 17: 17562864241243186.
11. Neuenschwander B, Capkun-Niggli G, Branson M, et al. Summarizing historical information on controls in clinical trials. *Clin Trials* 2010; 7: 5–18.
12. Röver C, Bender R, Dias S, et al. On weakly informative prior distributions for the heterogeneity parameter in Bayesian random-effects meta-analysis. *Res Synth Methods* 2021; 12: 448–474.
13. O’Hagan A, Forster J. *Kendall’s Advanced Theory of Statistics, volume 2B: Bayesian Inference*. 2nd ed. London: Arnold, 2004, p.480.
14. Schmidli H, Gsteiger S, Roychoudhury S, et al. Robust meta-analytic-predictive priors in clinical trials with historical control information. *Biometrics* 2014; 70: 1023–1032.
